# Supplementary material for: Does 3D-Assisted Operative Treatment of Pelvic Ring Injuries Improve Patient Outcome?—A Systematic Review of the Literature
Source: J Pers Med. 2021 Sep 18;11(9):930. doi: 10.3390/jpm11090930 (PMC8470452; doi:10.3390/jpm11090930)
Supplement: Supplementary file 1 [file jpm-11-00930-s001.zip › jpm-1334683-supplementary.pdf]

Supplemental Digital Content 1

Overview of 3D assisted pelvic ring injury surgery
Five applications of 3D technology in pelvic ring injury surgery were identified: '3D virtual fracture visualization', '3D printed model', 'pre-contouring of osteosynthesis material on a 3D printed model', '3D printed surgical guides', and 'intra-operative 3D imaging'. Some studies combined these techniques.

1) Pre-operative 3D virtual fracture visualization
Five studies [16,22,26,9,21] reported on the use of a virtual 3D model for visualization of the fracture(s). This is the basic method utilizing 3D in which the first step is 'segmentation', representing the process whereby CT data is processed. The pelvis is then identified and converted into a digital 3D model. Examples of software that allow for the production of these 3D models are Mimics Medical software (Materialise, Leuven, Belgium) or OrthoMap 3D (Stryker, Kalamazoo, MI, USA). A virtual 3D fracture model allows for detailed evaluation of the fracture pattern, implant selection and basic virtual surgical planning. Virtual screws can be superimposed on the 3D virtual model for pre-operative planning of screw positions, lengths and directions. Takao et al. [21] used the navigation unit of OrthoMap to plan screw positions, which can be adjusted before the actual surgery to keep a safety margin from the nerve root tunnels in S1 and S2. The virtual screws were subsequently superimposed on the 3D virtual model. Besides, Nie et al. [16] used Mimics software to determine the position of the osteosynthesis plate.

2) Pre-operative 3D printed model
Four studies [13,4,16,9] described the usage of a 3D printed model based on the CT scan of the injured pelvis. While 3D virtual models usually provide the surgeon with more information compared to regular CT imaging, 3D printed real-sized models provide surgeons with a tactile feedback of the volume, size and orientation of bone fragments. By understanding the fracture pattern, the optimal surgical approach, reduction technique, and screw trajectories can be planned. Depending on the used materials, some models can also be sterilized and brought to the surgical field. The models were used pre-operatively as a reference for surgeons to assess fracture characteristics and the degree of displacement. Besides, Li et al. [13] designed a 3D printed model including the arteries and veins. These 3D printed models were used to predetermine the optimal operative approach, fracture reduction strategy as well as shape and length of the plate. Besides, the 3D printed models in these studies were also used to predetermine the entry point, angle, amount and length of screws. This was done by simulating the surgery using actual screws. By using the contralateral healthy hemi-pelvis as a reference, Cai et al. [4] simulated the reduction procedure with Kirschner wires (K-wires). The K-wire was used to simulate implantation of the cannulated screw.

3) Pre-contouring of osteosynthesis material
Two studies [16,9] reported on the use of a 3D printed model for pre-contouring of osteosynthesis plates in order to improve implant positioning. To allow for this technique, a 3D printed mirrored image of the opposite intact hemi-pelvis is generated. The printed model is then used to determine the optimal implant sizes and length. Subsequently, the plate is contoured in order to optimally fit the patient-specific pelvic model. Nie et al. [16] also performed a simulated operation on the 3D model. Finally, the pre-contoured osteosynthesis plate is sterilized according to predefined protocols and applied during surgery.

4) 3D printed drilling guides

Two studies [26,5] designed and applied 3D printed surgical guides for sacroiliac screw placement. At the damage control phase an external fixator was applied and after initial stabilization a CT-scan was obtained for preoperative planning of definitive surgery. The external fixator pins were used as a reference to design a drilling guide for sacroiliac screw placement. A virtual 3D screw trajectory was simulated after which a drilling guide was designed in order to translate the virtual surgical plan to the actual surgery. Subsequently, the external templates were 3D printed using photosensitive resin material and sterilized for intra-operative use. After satisfactory fracture reduction during surgery, the drilling guide was firmly assembled to the external fixator pins. Subsequently, a K-wire was inserted through the drilling guide according to the preoperative plan. Standard fluoroscopic control was performed to verify the direction of the K-wire. Subsequently, the drilling guide was removed and a cannulated screw was inserted along the K-wire.

5) Intra-operative 3D imaging

The vast majority of the studies [2,6,3,20,10,14,18,7,22,23,1,21] used a 3D technique for intra-operative imaging. This was largely done during the placement of screws with a 3D fluoroscope or O-arm to verify the correct direction and depth of the guide wire pins or screws [10,7,22,23,1,3,20]. Other studies used the 3D fluoroscope or O-arm to verify the correct position after screw placement [2,18,1]. Using this technology, the surgeon will be able to make per-operative decisions based on 3D instead of 2D fluoroscopy images. In case of dissatisfaction with the fracture reduction or the position of the screw, the surgeon can decide immediately during the operation to perform a revision of the implant positions. Three studies [6,14,21] used 3D computer-assisted navigation of screws by using reference markers fixed near the surgical site, often the iliac crest, to relate anatomical locations and instrumentation. By matching these trackers with the navigation system, 3D real-time position of guides and screws could be visualized.
